# Efficient Production of Fc Fusion Proteins in the Cytoplasm of *Escherichia coli*: Dissecting and Mitigating Redox Heterogeneity

**DOI:** 10.3390/ijms232314740

**Published:** 2022-11-25

**Authors:** Aatir A. Tungekar, Lloyd W. Ruddock

**Affiliations:** Protein and Structural Biology Research Unit, Faculty of Biochemistry and Molecular Medicine, University of Oulu, 90220 Oulu, Finland

**Keywords:** *Escherichia coli*, CyDisCo system, Fc fusion proteins, redox heterogeneity, oxidative folding

## Abstract

Cost-effective production of therapeutic proteins in microbial hosts is an indispensable tool towards accessible healthcare. Many of these heterologously expressed proteins, including all antibody formats, require disulfide bond formation to attain their native and functional state. A system for catalyzed disulfide bond formation (CyDisCo) has been developed allowing efficient production of recombinant proteins in the cytoplasm of one of the most used microbial expression systems, *Escherichia coli*. Here, we report high-yield production (up to 230 mg/L from 3 mL cultures) of in-demand therapeutics such as IgG_1_-based Fc fusion proteins in the *E. coli* cytoplasm. However, the production of this drug class using the CyDisCo system faces bottlenecks related to redox heterogeneity during oxidative folding. Our investigations identified and addressed one of the major causes of redox heterogeneity during CyDisCo-based production of Fc fusion proteins, i.e., disulfide bond formation in the IgG_1_ C_H_3 domain. Here, we communicate that mutating the cysteines in the C_H_3 domain of target Fc fusion proteins allows their production in a homogeneous redox state in the cytoplasm of *E. coli* without compromising on yields and thermal stability.

## 1. Introduction

The success of biopharmaceutical industries is driven by time and cost-effective manufacturing of target recombinant proteins. There is no uncertainty that the production of recombinant proteins in microbial expression systems has revolutionized the domains of biochemistry and biotechnology [1]. *E. coli* has been a workhorse for recombinant protein production for over four decades and offers various advantages over other expression hosts such as comparatively easier genetic manipulation, cost-effective production, rapid cell growth, and a simple fermentation process [2,3]. One of the critical drawbacks of using wild type *E. coli* as an expression host is its inability to carry out post-translational modifications such as disulfide bond formation in the cytoplasm. However, the CyDisCo system offers an attractive solution for the high-yield production of disulfide-bonded recombinant proteins [4]. While a wide range of disulfide-bonded recombinant proteins has been successfully produced using the CyDisCo system, including scFv and Fab antibody fragments [5,6], production of commercially relevant modified antibody formats such as Fc fusion proteins has not been reported. Fc fusion proteins are chimeric proteins composed of a biologically active peptide or receptor domain fused to the fragment crystallizable (Fc) domain of an immunoglobulin G (IgG) antibody. Most of the molecules in this drug class have been produced using mammalian cell lines, while some are produced in *E. coli* in the form of insoluble aggregates that are subsequently refolded in vitro [7].

One rationale behind choosing to investigate the cost-effective production of candidates from the drug class of Fc fusion proteins is the ongoing demand to enhance the capabilities of therapeutic peptides [8]. The pharmacokinetic properties of therapeutic peptides such as low stability and fast renal clearance hamper their therapeutic application [9]. Grafting these peptides to the Fc region of an IgG offers many advantages such as decreased renal clearance owing to their increased size and the therapeutic activity being retained or even enhanced due to homodimerization which causes an increased avidity to the target (at least two peptides per molecule) [10]. Mammalian systems have been widely employed to produce therapeutic Fc fusion proteins and are often chosen over yeasts due to the importance of native glycosylation for FcRn interaction [11]. Although, Fc-mediated effector functions are not a part of the mechanism of action for some Fc fusion proteins and can hence be detrimental by causing off-mechanism toxicity [11]. For this class of therapeutics, efficient production in *E. coli* can be a desirable solution. Fc fusion proteins are homodimers involving the association of the Fc region of IgGs, and bottlenecks in their production in *E. coli* possibly arise due to the nature of the partner conjugated to the Fc region or inefficient soluble expression due to either non-native or absent disulfide bonds. The proteins of interest (POIs) in this research article have six to ten disulfides, and these multidisulfide-containing multidomain proteins seem to be good candidates for production using the CyDisCo system and for understanding the limitations of oxidative folding events in vivo.

Critical bottlenecks during biopharmaceutical process development that hinder the production of therapeutic proteins in a native state can be addressed by identifying the cause of disulfide-related heterogeneity. Here, we report high-yield production of IgG_1_-based Fc fusion proteins including hormone-Fc fusions and peptide-Fc fusions in the cytoplasm of *E. coli*. In addition, we also report the bottlenecks associated with redox heterogeneity of Fc fusion proteins as well as identify and address the major cause of redox heterogeneity when produced using the CyDisCo system.

## 2. Results and Discussion

### 2.1. Soluble Production of Fc Fusion Proteins in the E. coli Cytoplasm

The CyDisCo system employed in this study has been used as a single polycistronic plasmid-based system or more commonly as a dual-plasmid based system for the production of a range of disulfide-bonded recombinant proteins [12]. It involves the coexpression of a sulfhydryl oxidase (Erv1p from *S. cerevisiae*) and a protein disulfide isomerase (human PDI) in the cytoplasm of *E. coli* while leaving the natural reducing pathways intact. The combined action of these two redox catalysts aids oxidative folding and allows the soluble production of multidisulfide-containing recombinant proteins thereby circumventing the need for solubilization and in vitro refolding.

**Figure 1:**
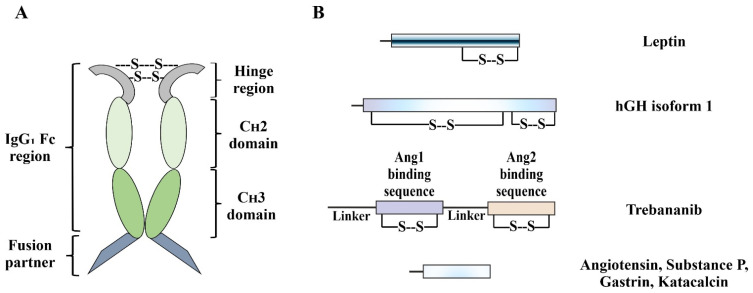
(**A**) Schematic representation of an IgG_1_-based Fc fusion protein. The IgG_1_ Fc domain is a natural homodimer consisting of three parts: the hinge region, C_H_2 domain and C_H_3 domain. Covalent interactions in the hinge region (intermolecular disulfides) as well as non-covalent interactions between the C_H_3 domains facilitate the homodimerization of the IgG_1_ Fc region. (**B**) Schematic representation of the fusion partners used in the study and their disulfide-bonding patterns (Ang1: Angiopoietin 1, Ang2: Angiopoietin 2). All the Fc fusion proteins used in this study have the partner hormone or peptide fused to the C-terminus of the C_H_3 domain.

We chose to test the expression of seven IgG_1_-based Fc fusion proteins which include two hormone-Fc fusions, namely, Leptin and Human Growth Hormone (hGH) isoform 1 and five Peptibodies, namely, Trebananib, Angiotensin–Fc, Substance P–Fc, Gastrin–Fc and Katacalcin–Fc (Figure 1).

**Figure 2:**
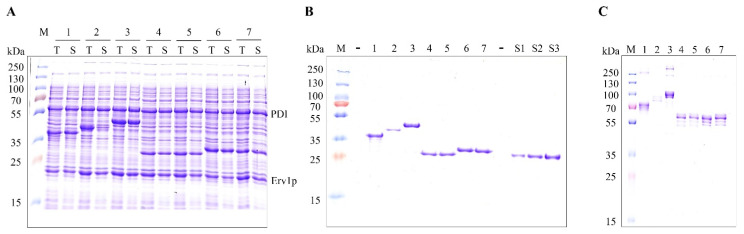
SDS-PAGE analysis of seven candidate Fc fusion proteins produced solubly with the help of the CyDisCo system in the cytoplasm of *E. coli* (**A**) SDS-PAGE gel image of T: total cell lysate and S: soluble cell lysate under reducing conditions. (**B**) SDS-PAGE gel image of Protein G-based purified POIs and purified wild type IgG_1_ Fc region in three different concentrations under reducing conditions. (**C**) SDS-PAGE gel image of Protein G-based purified POIs under non-reducing and N-Ethyl maleimide (NEM)-treated conditions showing redox heterogeneity. M: marker, 1: Trebananib, 2: Leptin–Fc, 3: hGH–Fc, 4: Angiotensin–Fc, 5: Substance P–Fc, 6: Gastrin–Fc, 7: Katacalcin–Fc, S1: IgG_1_ Fc region (0.19 µg), S2: IgG_1_ Fc region (0.38 µg), S3: IgG_1_ Fc region (0.56 µg).

The first step was to investigate whether the chosen Fc fusion proteins could be produced solubly in the *E. coli* cytoplasm with the help of CyDisCo components. All the POIs tested were produced solubly (Figure 2A) and purified efficiently (Figure 2B) owing to the binding affinity of the Fc region of the fusion protein to Protein G. These target proteins were found to be produced in remarkably high yields from 3 mL cultures (Table 1). It can be observed in Figure 2A that the Leptin–Fc fusion is not very soluble as compared to the total amount of protein produced (Lane 2), which correlates with its comparatively lower purified yields (Table 1). The range of yields obtained for the target POIs suggest that the partner protein or peptide fused to the IgG_1_ Fc region has a significant influence on the solubility of the fusion protein and/or its folding intermediates.

However, soluble production does not guarantee that the recombinant protein is natively folded and is produced in a homogeneous redox state [13]. Although disulfide bond formation is essential for protein folding, stability and function, some proteins can remain in the soluble state even in the absence of native disulfides. It can be observed that most of the proteins produced show a single band on an SDS-PAGE gel under reducing conditions, but multiple bands under non-reducing conditions suggest disulfide-related heterogeneity, i.e., they are produced in multiple redox states (Figure 2C). In contrast, Trebananib (AMG-386) was found to be prone to degradation in the cytoplasm of *E. coli* as seen in the multiple purified protein bands under reducing conditions (Figure 2B). To minimize proteolysis, an additional experiment was set up using a protease-deficient strain *E. coli* MDS42 [14] (Figure S1). Once negligible proteolysis was ensured, Trebananib was found to exhibit the same redox heterogeneity as the other POIs.

It is important to investigate redox heterogeneity as one of the critical quality attributes during recombinant protein production, as previous studies have indicated that misfolded proteins can be a potential cause of immunogenicity [15]. Hence, building quality in the final product can only be achieved by reducing misfolded isomers of therapeutic proteins, as one of the protein-related impurities.

### 2.2. Identifying the Cause of Redox Heterogeneity in Fc Fusion Proteins

The CyDisCo system has been successfully employed for the production of a 44-disulfide containing highly complex ECM (extracellular matrix) protein in a natively folded and homogeneous redox state [13], suggesting that the redox heterogeneity observed with Fc fusion proteins might arise due to the nature of the protein instead of a limitation of the redox catalysts. Prior to identifying the major cause of redox heterogeneity, it is essential to understand that the fusion partners in some of the Fc fusion proteins, mainly Peptibodies, i.e., Angiotensin, Gastrin, Substance P, and Katacalcin do not contain any cysteines and hence are not the cause of the heterogeneous redox states (Figure 1). Owing to the fact that these aforementioned POIs are still produced in multiple redox forms, we focused our attention on the part of the fusion protein that contains cysteines and hence needs disulfide bond formation, i.e., the Fc region of IgG_1_. A typical IgG_1_ molecule is a homodimer consisting of 16 disulfides with 10 disulfides in the Fab region of the antibody and 6 disulfides in the Fc region. The distribution of these covalent interactions in the Fc region of an IgG_1_ consists of two intermolecular disulfides in the hinge region and two intramolecular disulfides in each of the C_H_2 and C_H_3 domains. The C_H_3 domains tightly pack with each other in a homodimeric Fc through multiple non-covalent interactions on the complementary faces (Figure 1), while the C_H_2 domains are more flexible and have no observable protein–protein contacts with each other [16].

**Figure 3:**
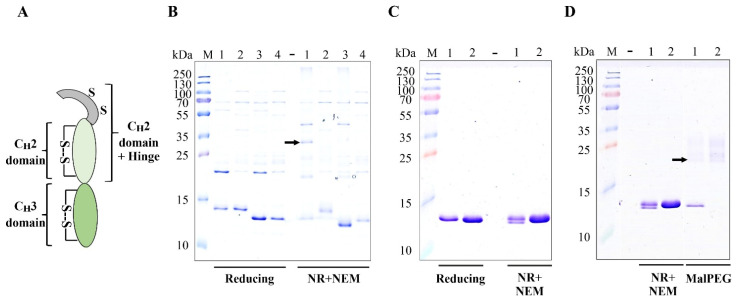
(**A**) Schematic representation of individual domains expressed to identify the cause of redox heterogeneity. (**B**) SDS-PAGE gel image of IgG_1_ C_H_2 domain with and without hinge region expressed in the presence and absence of CyDisCo. A dimer can be observed (marked with an arrow) under non-reducing (NR), NEM-treated conditions for the IgG_1_ C_H_2 domain with hinge region (Lane 1) in the presence of CyDisCo corresponding to the intermolecular disulfide in the hinge region. M: protein marker, 1: IgG_1_ C_H_2 domain with hinge + CyDisCo, Lane 2: IgG_1_ C_H_2 domain with hinge–CyDisCo, Lane 3: IgG_1_ C_H_2 domain + CyDisCo, Lane 4: IgG_1_ C_H_2 domain–CyDisCo. (**C**) SDS-PAGE gel image of the IgG_1_ C_H_3 domain expressed in the presence and absence of CyDisCo. The gel shows redox heterogeneity for the IgG_1_ C_H_3 domain in the presence of CyDisCo under non-reducing (NR), NEM-treated conditions. M: protein marker, Lane 1: + CyDisCo, Lane 2:– CyDisCo. (**D**) MalPEG-5K (0.25 mM) assay to ascertain the redox state of IgG_1_ C_H_3 domain produced in the presence and absence of CyDisCo. Mobility shift observed due to MalPEG binding has been marked with an arrow. M: protein marker, Lane 1: + CyDisCo, Lane 2:– CyDisCo.

To ascertain which cysteines or disulfides in the IgG_1_ Fc region cause redox heterogeneity, we investigated the expression of different domains of the IgG_1_ Fc region. These included the hinge region fused to the C_H_2 domain, the individual C_H_2 domain and the individual C_H_3 domain (Figure 3A).

These domains were expressed in the cytoplasm of *E. coli* in the presence and absence of CyDisCo to identify their redox states as well as to understand the effect of disulfide bond formation on soluble expression. As clearly observed by the migration shifts under reducing and non-reducing conditions, the CyDisCo system catalyzes disulfide bond formation in the C_H_2 domains with (T106-A222) and without the hinge region (G119-A222) (Figure 3B). In addition, the C_H_2 domain without the hinge region is produced in higher yields in the presence of CyDisCo suggesting that disulfide bond formation contributes to its solubility via allowing it to attain its native state. In the absence of redox catalysts, the migration pattern of the two constructs remains identical under reducing and non-reducing conditions suggesting the absence of disulfide bonds. Although these proteins are produced in very low soluble yields, they are produced in a single homogeneous redox state.

In contrast, the C_H_3 domain is produced solubly and in comparatively higher yields in the absence of CyDisCo suggesting that it can fold even in the absence of its intramolecular disulfide bond (Figure 3C). It has been shown that the tertiary and quaternary structure of the reduced C_H_3 domain remains unchanged as compared to the native, oxidized state [17]. This is in accordance with our results and the high soluble yields observed. However, under non-reducing, NEM-treated conditions, it can be clearly observed that the C_H_3 domain is produced in two redox states in the presence of CyDisCo (Lane 1, Figure 3C). As the C_H_3 domain has one intramolecular disulfide bond, the SDS-PAGE analysis reveals that the redox catalysts can partially catalyze disulfide bond formation in the C_H_3 domain.

To further investigate, we carried out a MalPEG assay in order to understand the redox status of the IgG_1_ C_H_3 domains produced in the presence and absence of CyDisCo. MalPEG-5K is an alkylating agent, i.e., it reacts with sulfhydryl (thiol/SH) groups and is a PEG derivative used to selectively modify proteins with available sulfhydryl groups. Once MalPEG forms a covalent bond with the free sulfhydryl groups on a protein, the MalPEG–protein conjugate can be detected as a band shift (~5 kDa per free thiol) on SDS-PAGE [18]. As evident from Figure 3D, C_H_3 domain produced in the absence of CyDisCo does not contain any disulfide bonds, i.e., it has free sulfhydryl groups available to react with MalPEG. This covalent interaction with MalPEG resulted in a complete upwards shift in the migration pattern. However, of the two redox states produced in the presence of CyDisCo, the reduced C_H_3 domain which lacks the disulfide bond was found to migrate upwards on the gel while the oxidized C_H_3 domain did not show a mobility shift as it has no free sulfhydryl groups available to interact with the MalPEG molecule. This confirmed that the C_H_3 domain was produced in two redox states.

The inability of the redox catalysts to form a completely oxidized C_H_3 domain suggests that either the thiol groups might not be accessible to the redox catalysts, i.e., they are buried in the core of the domain or the domain folds prior to disulfide bond formation, hence making the thiols inaccessible for oxidation. Previous refolding/oxidation experiments of the C_H_3 domain have indicated that there is an intrinsic complexity in disulfide formation in the structured C_H_3 domain due to steric constraints [17]. Their findings suggest that folding and oxidation of the C_H_3 domain are tightly coupled such that formation of a stable tertiary structure decelerates redox shuffling due to the burial of the reactive thiol groups [17]. This combined with our results confirms our hypothesis that the redox heterogeneity arises due to the nature of the POIs. It also suggests that the disulfides in the C_H_3 domain are possibly the major cause of redox heterogeneity observed in CyDisCo-based Fc fusion protein production in the cytoplasm of *E. coli*.

### 2.3. Mitigating the Cause of Redox Heterogeneity in Fc Fusion Proteins

Unfolded and misfolded protein isoforms can contribute to the pathology of various diseases. Hence, production of proteins in a homogeneous and natively folded state either by in vitro refolding or oxidative folding in vivo, remains an obstacle for protein-based therapeutics. It has been demonstrated in serum antibodies of healthy donors that the IgG_1_ molecule contains free sulfhydryl (SH-) groups and disulfide (-S–S-) bonds simultaneously, which raises a question on the role of cysteine residues in the functioning of antibodies as well as in the structure of an IgG [19,20]. Although the absence of disulfide bonds in immunoglobulin (Ig) domains does not restrict its folding to an active molecule, free cysteines have only been identified after denaturation, suggesting that the cysteines which should be in intramolecular disulfides buried within the Ig domains can exist in a reduced state [21]. Further analysis of recombinantly produced monoclonal antibodies has revealed a higher percentage of missing disulfides in the C_H_3 domain [20,22].

**Figure 4:**
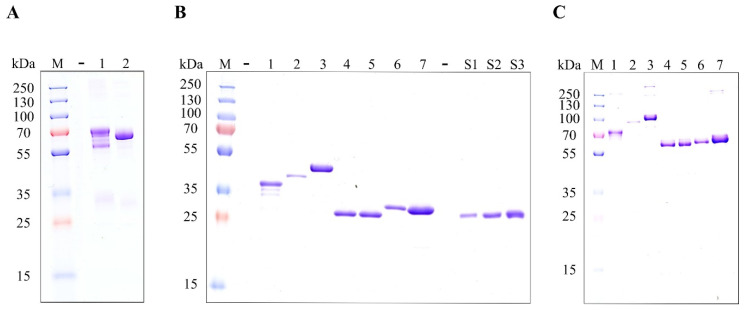
(**A**) SDS-PAGE analysis of wild type and mutant IgG_1_ Fc region under non-reducing and NEM-treated conditions showing that the mutating the cysteines in the C_H_3 domain to alanine results in the production of the IgG_1_ Fc region in a single homogeneous redox state. M: protein marker, 1: IgG_1_ Fc region wild type, Lane 2: IgG_1_ Fc region (C250A, C308A). (**B**) SDS-PAGE gel image of Protein G-based purified POIs with a mutant IgG_1_ Fc region (C250A, C308A) and purified wild type IgG_1_ Fc region in three different concentrations under reducing conditions. (**C**) SDS-PAGE gel image of Protein G-based purified POIs with a mutant IgG_1_ Fc region (C250A, C308A) under non-reducing and NEM-treated conditions showing that the fusion proteins are produced in a single homogeneous redox state. M: marker, 1: Trebananib, 2: Leptin–Fc, 3: hGH–Fc, 4: Angiotensin–Fc, 5: Substance P–Fc, 6: Gastrin–Fc, 7: Katacalcin–Fc, S1: IgG_1_ Fc region (0.19 µg), S2: IgG_1_ Fc region (0.38 µg), S3: IgG_1_ Fc region (0.56 µg).

After empirical identification of the potential cause of redox heterogeneity, we mutated the cysteines in the C_H_3 domain to alanine (C250A, C308A–nomenclature of immunoglobulin heavy constant gamma 1, UniProt: P01857) in order to investigate how it influences the redox status of the IgG_1_ Fc region when produced using CyDisCo in the cytoplasm of *E. coli*. We hypothesized that the absence of free thiols in the C_H_3 domain mitigates the need for redox shuffling during folding, and hence redox heterogeneity. As observed in Figure 4A, under non-reducing and NEM-treated conditions, the wild type IgG_1_ Fc region is produced in multiple redox states while the IgG_1_ Fc region with the cysteines in the C_H_3 domain mutated to alanine is produced in a single homogeneous redox state. This observation confirms our hypothesis that the redox heterogeneity in IgG_1_ based Fc fusion protein production arises mainly from the disulfides in the C_H_3 domain.

These findings were then applied to Fc fusion protein candidates with cysteines in the C_H_3 domain of the Fc region mutated to alanine. Figure 4B shows that these Fc fusion protein mutants can be produced solubly and purified efficiently without any major compromise on the yields (Table 2). More importantly, we demonstrate here that these Fc fusion protein mutants can be produced in a single homogeneous redox form (Figure 4C) in remarkably high yields from 3 mL cultures. It can also be observed that mutating the cysteines in the C_H_3 domain of these Fc fusion proteins does not interfere with the homodimerization of these proteins (Figure 4C). To the best of our knowledge, this is the first investigation that reports such high yields of Fc fusion proteins produced in a homogeneous redox state in the cytoplasm of *E. coli*.

### 2.4. Effect of Mutations on Protein Stability

**Figure 5:**
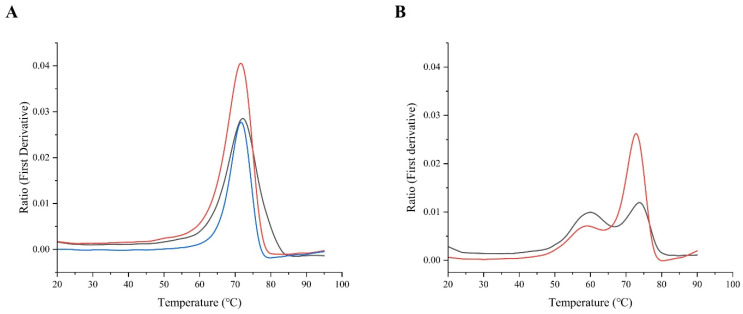
Thermal stability of purified proteins using Nano Differential Scanning Fluorimetry (nanoDSF). (**A**) Thermal stability of partially oxidized wild type IgG_1_ C_H_3 domain produced with CyDisCo (black), reduced wild type IgG_1_ C_H_3 domain (red) and IgG_1_ C_H_3 domain (C250A, C308A) (blue). A single unfolding event can be observed at the same temperature suggesting no influence of the mutation on thermal stability or dimerization of the C_H_3 domain. (**B**) Thermal stability of wild type IgG_1_ Fc region (black) and IgG_1_ Fc region (C250A, C308A) (red). Two thermal unfolding events can be observed with the peak at the higher temperature corresponding to the unfolding of the C_H_3 domain. No significant influence of the mutation on thermal stability was observed.

In addition to obtaining the protein in a homogeneous redox state, we also investigated the effect of the mutations on the thermal stability of the IgG_1_ C_H_3 domain and IgG_1_ Fc region through thermal unfolding experiments using Nano Differential Scanning Fluorimetry (NanoDSF). As the dimerization interface of the C_H_3 domain remains intact even in the absence of an intramolecular disulfide [17], the C_H_3 homodimer is a highly stable complex. The partially oxidized IgG_1_ C_H_3 dimer produced in the presence of CyDisCo does not show any major shift in thermal stability between oxidized and reduced species (Figure 5A). Our findings demonstrate that the IgG_1_ C_H_3 dimer is a highly thermostable protein in isolation even under reducing conditions and mutating its cysteines to alanine does not alter its thermal stability (Table 3). Thermal unfolding experiments of the IgG_1_ Fc domain show two unfolding events corresponding to the C_H_2 and C_H_3 domains (Figure 5B). The peak at a higher temperature in Figure 5B corresponds to the thermal unfolding of the C_H_3 domain which is in accordance with earlier reports that the IgG C_H_3 domain has a higher thermal stability than the C_H_2 domain [23]. Our findings suggest that mutating the cysteines of the C_H_3 domain to alanine does not have a significant influence on the thermal stability of the IgG_1_ Fc domain when produced using CyDisCo in the cytoplasm of *E. coli* (Table 3).

## 3. Materials and Methods

### 3.1. Cloning

Expression vectors (see Table S1 for vectors used in this study) were constructed using standard molecular biology techniques. Genes for human IgG_1_ Fc wild type and IgG_1_ Fc region (C250A, C308A) having a C-terminal hexahistidine flanked by two BamHI sites were synthesized via codon optimization (GenScript Biotech Corp., Piscataway, NJ, USA) for *E. coli* expression. These two genes were cloned using restriction digestion and ligation into a modified pET23-based vector with a pTac promoter replacing the T7 promoter [5] to generate vectors pAAT44 and pAAT81, respectively. Genes for Trebananib fusion partner and Leptin with BamHI-EcoRI flanking sites were synthesized via codon optimization (GenScript Biotech Corp.) for *E. coli* expression. Angiotensin, Substance P, Gastrin and Katacalcin peptides were synthesized as complementary single-stranded oligonucleotides with BamH1-EcoR1 overhangs (Table S2). These oligonucleotides were annealed by heating to 95 °C and gradual cooling to 25 °C with a ramp down of 1 °C/min. Human growth hormone isoform 1 (F27-F217) gene was amplified using PCR from pYU7 with primers designed to have BamHI-EcoRI flanking sites. The IgG_1_ Fc region wild type and IgG_1_ Fc region (C250A, C308A) genes were digested and ligated using BamHI to remove the C-terminal hexahistidine tag to generate vectors pAAT115 and pAAT85, respectively. All the fusion partner genes were cloned using restriction digestion and ligation into vectors pAAT115 and pAAT85 using BamH1-EcoR1 to generate Fc fusion protein genes with the partner protein/peptide fused at the C-terminus of IgG_1_ Fc region. The IgG_1_ C_H_3 domain (C250A, C308A) gene was amplified using PCR from pAAT81 to have a C-terminal hexahistidine tag and cloned into the same backbone vector [5] using restriction enzyme pairs NdeI-EcoRI. Genes for the IgG_1_ C_H_2 domain with the hinge region (T106-A222) and the IgG_1_ C_H_2 domain without the hinge region (G119-A222) were amplified using PCR from pAAT44 and cloned into a pET23-based vector with a pTac promoter replacing the T7 promoter (pMJS162) using restriction enzyme pairs NdeI-BamH1 such that it has an N-terminal hexahistidine tag prior to the first amino acid of the protein sequence. Sequences of all the primers and single-stranded complementary oligonucleotide pairs used in this study have been included in Table S2. All the plasmids were purified using the E.Z.N.A. Plasmid DNA Mini Kit I (Omega Bio-Tek Inc., Norcross, GA, USA) and all purification from agarose gels was performed using the Gene/PCR DNA Fragments Extraction Kit (GeneAid Biotech, Sintai, Taiwan), both according to the manufacturers’ guidelines. All the gene inserts in the constructed vectors were fully sequenced prior to use to avoid any errors in the cloned genes.

### 3.2. Protein Expression

Plasmids containing the genes of interest along with the polycistronic plasmid containing CyDisCo components Erv1p and PDI (pMJS205) [5] were used to co-transform chemically competent *E. coli* BL21(DE3) strains (Stratagene) and allowed to grow overnight at 37 °C on Lysogeny Broth (LB) agar plates supplemented with appropriate antibiotics for selection (35 µg/mL chloramphenicol for pLysS derivatives and 100 µg/mL ampicillin for pET23 derivatives). Selected transformants from the plates were used to inoculate 2 mL of LB media supplemented with 2 g/L glucose and suitable antibiotics, and the cultures were grown at 30 °C, 250 rpm (2.5 cm radius of gyration) in 24 deep well plates covered with oxygen permeable AirOTop (Thomson) membranes for 6–8 h. These starter cultures were used to seed expression cultures containing 3 mL terrific broth autoinduction media (Formedium) filtered with 0.2 µm membrane filters and supplemented with 0.8% glycerol and suitable antibiotics per well in a 1:100 ratio. These expression cultures were grown at 30 °C, 250 rpm in 24 deep well plates covered with oxygen permeable AirOTop (Thomson) membranes to ensure efficient oxygenation for approximately 23–24 h. Final optical density values of the cultures were measured at 600 nm and were found to be in the range of 16.5–20.0. The cultures were harvested by centrifugation at 6500× *g* at 4 °C and the cell pellets were resuspended in 3 mL of lysis buffer containing 50 mM sodium phosphate pH 7.4, 20 μg/mL DNase, and 0.1 mg/mL egg white lysozyme. The resuspended cultures were incubated for 15 min at room temperature and frozen at −20 °C. Cells were lysed by freeze–thawing.

For the expression of the IgG_1_ Fc region wild type to be used as a standard for densitometric analysis, selected transformants of *E. coli* BL21(DE3) containing the plasmid of interest (pAAT44) and the CyDisCo plasmid (pMJS205) were used to inoculate 10 mL of LB media supplemented with 2 g/L glucose and suitable antibiotics, and the cultures were grown at 30 °C, 250 rpm (2.5 cm radius of gyration) in 100 mL flasks covered with oxygen permeable AirOTop (Thomson) membranes for 6–8 h. These starter cultures were used to seed expression cultures containing 100 mL terrific broth autoinduction media (Formedium) filtered with 0.2 µm membrane filters and supplemented with 0.8% glycerol and suitable antibiotics in a 1:100 ratio. These expression cultures were grown at 30 °C, 250 rpm in 1 L flasks covered with oxygen permeable AirOTop (Thomson) membranes to ensure efficient oxygenation for approximately 23–24 h. Cells were harvested by centrifugation at 6500 × *g* at 4 °C and the cell pellets were frozen at −20 °C.

### 3.3. Protein Purification

#### 3.3.1. Protein G-Based Purification

Purification of the IgG_1_ Fc region wild type and mutant (C250A, C308A), as well as all candidate Fc fusion proteins was carried out using a Protein G Sepharose™ 4 Fast Flow (GE Healthcare) resin under native conditions without using any additional affinity tags. The cell lysate was clarified by centrifugation (4000 rpm, 20 min, 4 °C). For 3 mL cultures from a 24 deep well plate, protein purification was performed using 0.75 mL of resin in small Poly-Prep^®^ gravity feed columns (Bio-Rad). The resin was washed with 2 × 5 mL of water, equilibrated with 2 × 5 mL of 20 mM phosphate buffer (pH 7.0). After loading the sample, the column was equilibrated with 2 × 2.5 mL of 20 mM phosphate buffer (pH 7.0), washed with 2 × 2.5 mL of wash buffer (20 mM sodium phosphate, 0.4 M sodium chloride; pH 7.0) followed by 5 mL of 20 mM sodium phosphate (pH 7.0). The bound proteins were eluted with 4 × 0.5 mL of elution buffer (0.1 M Glycine-HCl, pH 3.0) into tubes containing 200 µL of neutralization buffer (1 M Tris; pH 9.0).

#### 3.3.2. Cobalt–IMAC-Based Purification

Proteins of interest containing a hexahistidine tag were purified with standard immobilized metal affinity chromatography (IMAC) using HisPur Cobalt Superflow Agarose (Thermo Scientific, Waltham, MA, USA) resin under native conditions following clearance of the cell lysate by centrifugation (4000 rpm, 20 min, 4 °C). For 3 mL cultures from a 24 deep well plate, protein purification was performed using 0.25 mL of resin in small Poly-Prep^®^ gravity feed columns (Bio-Rad). The resin was washed with 2 × 5 mL of water, equilibrated with 2 × 5 mL of 50 mM phosphate buffer (pH 7.4). After loading the sample, the column was equilibrated with 2 × 2.5 mL of 50 mM phosphate buffer (pH 7.4), and washed with 4 × 2.5 mL of wash buffer (50 mM sodium phosphate, 15 mM Imidazole, 0.3 M sodium chloride; pH 7.4) followed by 5 mL of 50 mM sodium phosphate (pH 7.4). The bound proteins were eluted with 4 × 0.25 mL of elution buffer (50 mM sodium phosphate, 50 mM EDTA; pH 7.4).

#### 3.3.3. Purification of Wild–Type IgG_1_ Fc Region

The wild type IgG_1_ Fc region having a C-terminal hexahistidine tag was purified using a two-step chromatography approach. The cell pellets were resuspended in 100 mL of lysis buffer containing 50 mM sodium phosphate pH 7.4, 20 μg/mL DNase, and 10 mM Imidazole. Cell lysis was carried out using sonication for 18 cycles of 5 s pulse on, 20 s pulse off at 70% amplitude. The cell lysate was centrifuged (14,500 rpm, 30 min, 4 °C) and the soluble fraction was filtered using a 0.45 µm membrane filter.

The first step of purification was carried out using nickel IMAC. Nickel was loaded onto a HiTrap™ 5 mL chelating HP column (GE Healthcare, Little Chalfont, UK) using 0.1 M Nickel chloride. The charged column was then washed with 10 column volumes (CV) of millipore water followed by equilibration using 20 mM sodium phosphate; pH 7.4. The soluble fraction was loaded onto the Ni-IMAC column at a 2 mL/minute flow rate followed by 4 CV of equilibration buffer. The loaded column was then washed with 20 mM sodium phosphate, 50 mM imidazole, 150 mM sodium chloride; pH 7.4, followed by 4 CV of equilibration buffer. The bound proteins were eluted using a linear (10 CV) gradient of 20 mM sodium phosphate, 300 mM Imidazole, 150 mM sodium chloride; pH 7.4. The second step of purification was Anion Exchange (AnEx) chromatography using a Resource Q™ column (GE Healthcare). The column was washed with 5 CV of Millipore water followed by equilibration using 5 CV of 20 mM Tris; pH 9.0. The eluted fractions were desalted using a 10 kDa cut off centrifugal filter (Merck), diluted 10× using the equilibration buffer and loaded onto the column at a 2 mL/minute flow rate. The bound proteins were eluted using a linear (10 CV) gradient of 20 mM Tris, 1 M sodium chloride; pH 9.0. The eluted fractions were then analyzed using SDS-PAGE under reducing conditions. The fractions containing the purified protein of interest were then pooled, concentrated, and used as a standard (>99% purity) for densitometric analysis of Fc fusion protein yields.

### 3.4. Protein Analysis

SDS-PAGE analysis of reduced (β-mercaptoethanol) and non-reduced, NEM trapped samples was carried out using 12.5% SDS-PAGE gels or 4–20% Criterion™ TGX™ Precast Midi Protein Gel (Bio-Rad). Appropriate samples were treated with 25 mM NEM at room temperature for 10 min prior to addition of SDS loading buffer. The proteins were detected using Coomassie Blue R-250 following electrophoretic separation. The purified protein yields were calculated in triplicates based on densitometric analysis using purified wild type IgG_1_ Fc region as a standard in three different concentrations. Densitometric analysis to determine target protein yields was carried out using the ImageJ software.

For the MalPEG-5K assay, the eluted samples were diluted 3× with 50 mM sodium phosphate; pH 7.4 to a final volume of 100 µL. This was followed by the addition of 10 µL of 1 M Tris; pH 8.0, 5 µL of 2% SDS, and 30 µL of non-reducing SDS loading buffer. This mixture was heated at 95 °C for 5 min to allow for protein denaturation and accessibility to buried thiols. The samples were allowed to cool for 2–3 min, treated with 5 µL of 7.5 mM MalPEG-5K (Sigma-Aldrich) and allowed to incubate at room temperature for 45 min. The samples were then used for SDS-PAGE analysis to ascertain the redox state of the proteins of interest.

Thermal stability analysis was carried out by Nano Differential Scanning Fluorimetry (nanoDSF) using Prometheus NT.48 (NanoTemper Technologies, Munich, Germany). The purified proteins were buffer exchanged into 20 mM sodium phosphate; pH 7.4 using Zeba™ Spin Desalting Columns (Thermo Scientific) and loaded into nanoDSF grade standard capillaries (10 µL). The samples containing capillaries were then subjected to programmed heating from 20 °C to 90 °C by a thermal ramping rate of 1 °C/min. The fluorescence signal resulting from the thermal unfolding of the protein was measured at 330 nm and 350 nm with a dual UV detector. The curve was generated using the first derivative of the ratio between the two signals to determine the unfolding transition midpoint (inflection point of the underlying ratio curve). The melting temperature (Tm) was calculated by PR.ThermControl software (NanoTemper Technologies). All thermal stability experiments were performed in triplicates.

## 4. Conclusions

The results presented in this study provide a significant proof-of-concept towards the applicability of the CyDisCo system for the efficient production of high-demand therapeutics such as modified antibody formats. We report high-yield soluble production of seven IgG_1_-based Fc fusion protein candidates for various therapeutic applications in the cytoplasm of *E. coli*. Our findings suggest that CyDisCo-based production of these molecules leads to their production in multiple redox states. We have empirically identified that the redox heterogeneity arises from a difficulty in disulfide bond formation in the C_H_3 domain of an IgG_1_ Fc, a feature of its structure, and not due to a fault of the redox catalysts. We found that mutating the cysteines in the C_H_3 domain to alanine addresses the bottleneck of redox heterogeneity and allows the production of all Fc fusion candidates tested in a single redox state without compromising on target protein yield and thermal stability. We believe such high-yield production of candidates from this drug class in a homogeneous redox form broadens the horizon of therapeutic antibody production in the cytoplasm of *Escherichia coli*.

## Figures and Tables

**Table 1:** Yields of wild type Fc fusion proteins purified from 3 mL cultures (24 DWP).

Fc Fusion Proteins (Wild Type)	Purified Yields (mg/L)
Trebananib	178 ± 7
Leptin–Fc	61 ± 4
hGH–Fc	196 ± 2
Angiotensin–Fc	148 ± 5
Substance P–Fc	138 ± 8
Gastrin–Fc	142 ± 4
Katacalcin–Fc	131 ± 1

**Table 2:** Yields of mutant Fc fusion proteins (C250A, C308A) purified from 3 mL cultures (24 DWP).

Fc Fusion Proteins (Mutants)	Purified Yields (mg/L)
Trebananib	117 ± 4
Leptin–Fc	37 ± 4
hGH–Fc	216 ± 2
Angiotensin–Fc	157 ± 4
Substance P–Fc	221 ± 5
Gastrin–Fc	105 ± 5
Katacalcin–Fc	230 ± 4

**Table 3:** Thermal stability analysis of wild type and mutant IgG_1_ C_H_3 domain and IgG_1_ Fc region using NanoDSF.

Protein of Interest (POI)	Melting Temperatures (Tm) (°C)
**IgG_1_ C_H_3 domain**	
Wild type (+CyDisCo)	72.1 ± 0.1
Wild type (-CyDisCo)	71.6 ± 0.1
Mutant (C250A, C308A)	71.5 ± 0.1
**IgG_1_ Fc region**	
Wild type	59.9 ± 0.1; 73.7 ± 0.1
Mutant (C250A, C308A)	59.6 ± 1.1; 72.9 ± 0.2

## Data Availability

The data presented in this study is contained within the article and Supplementary Material.

## Supplementary Materials

The supporting information can be downloaded at: https://www.mdpi.com/article/10.3390/ijms232314740/s1.

## Abbreviations

DWP	deep well plate
NEM	N-Ethyl maleimide
MalPEG	Methoxypolyethylene glycol maleimide
IgG	immunoglobulin G
Fc	crystallizable fragment
FcRn	Neonatal Fc receptor
IMAC	immobilized metal affinity chromatography
EDTA	Ethylenediaminetetraacetic acid
