# Supplementary material for: Efficient Production of Fc Fusion Proteins in the Cytoplasm of Escherichia coli: Dissecting and Mitigating Redox Heterogeneity
Source: Int J Mol Sci. 2022 Nov 25;23(23):14740. doi: 10.3390/ijms232314740 (PMC9737693; doi:10.3390/ijms232314740)
Supplement: Supplementary file 1 [file ijms-23-14740-s001.zip › ijms-2021778-supplementary.pdf]

Supporting information

1. Trebananib production in *E. coli* MDS42

Trebananib constructs containing the wild-type IgG_1 Fc and IgG_1 Fc (C250A, C308A) were expressed in a multi-protease deficient strain *E. coli* MDS42 to examine whether the multiple bands observed under non-reducing, NEM treated conditions arise due to redox heterogeneity and not as a result of proteolysis. The plasmids containing the genes of interest (pAAT57 or pAAT87) and the CyDisCo plasmid (pMJS205) were used to cotransform *E. coli* MDS42. The expression and culture harvesting were carried using the same protocol as described except that the lysis buffer used for this test contained Pierce™ EDTA-free protease inhibitor tablets (1 tablet/50 ml) (Thermo Scientific). The resuspended cultures were allowed to incubate for 15 minutes at room temperature and frozen at -20°C. Cells were lysed by freeze-thawing. The proteins of interest in the soluble cell lysate were purified using Protein-G as per the protocol and analyzed using SDS-PAGE. Appropriate samples were treated with 25 mM NEM at room temperature for 10 minutes prior to addition of SDS loading buffer.

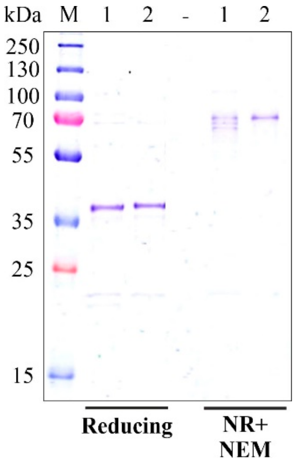

Figure S1. SDS-PAGE analysis of Trebananib with wild-type IgG_1 Fc region and IgG_1 Fc region (C250A, C308A) produced in *E. coli* MDS42 and cells lysed in the presence of protease inhibitors to significantly reduce proteolysis. The gel image shows that the wild type Trebananib shows redox heterogeneity while the mutant Trebananib is produced in a single homogeneous redox state under non-reducing (NR), NEM treated conditions. M: Protein marker, Lane 1: Trebananib wild type, Lane 2: Trebananib with mutant IgG_1 Fc (C250A, C308A).

Table S1. Details of plasmid vectors used in the study

Construct details	Plasmid name	References
tPA kringle, N-terminal His6 tag	pMJS162	[5]
Growth hormone 1 isoform 1 (F27-F217F)	pYU7	This study
Trebananib peptide (G1-E59)	pAAT41	This study
Leptin (V1-C146)	pAAT43	This study
IgG_1 Fc region (S1-S229), C-terminal His6 tag	pAAT44	This study
IgG_1 Fc region (S1-S229)	pAAT115	This study
Trebananib with IgG_1 Fc wild type	pAAT57	This study
Leptin - IgG_1 Fc wild type fusion	pAAT59	This study
hGH isoform 1 - IgG_1 Fc wild type fusion	pAAT61	This study
Angiotensin - IgG_1 Fc wild type fusion	pAAT65	This study
SubstanceP - IgG_1 Fc wild type fusion	pAAT67	This study
Gastrin - IgG_1 Fc wild type fusion	pAAT69	This study
Katacalcin - IgG_1 Fc wild type fusion	pAAT71	This study
IgG_1 C_H2 domain with hinge (T106-A222), N-terminal His6 tag	pAAT72	This study
IgG_1 C_H2 domain without hinge (G119-A222), N-terminal His6 tag	pAAT74	This study
IgG_1 C_H3 domain, C-terminal His6 tag	pYU315	This study
IgG_1 C_H3 domain (C250A, C308A), C-terminal His6 tag	pAAT117	This study
IgG_1 Fc region (C250A, C308A), C-terminal His6 tag	pAAT81	This study
IgG_1 Fc region (C250A, C308A)	pAAT85	This study
Trebananib with IgG_1 Fc (C250A, C308A)	pAAT87	This study
Leptin - IgG_1 Fc (C250A, C308A) fusion	pAAT89	This study
hGH isoform 1 - IgG_1 Fc (C250A, C308A) fusion	pAAT91	This study
Angiotensin - IgG_1 Fc (C250A, C308A) fusion	pAAT93	This study
SubstanceP - IgG_1 Fc (C250A, C308A) fusion	pAAT95	This study
Gastrin - IgG_1 Fc (C250A, C308A) fusion	pAAT97	This study
Katacalcin - IgG_1 Fc (C250A, C308A) fusion	pAAT99	This study
CyDisCo components	pMJS205	[5]
CyDisCo negative control	pAG82	[5]

Table S2. Details of primers used in the study

Primer	Sequence
**Complementary oligonucleotides for peptibodies**	
Angiotensin - Forward	GATCGGACCGGGTGTATATTCATCCGTTTCATCTGTAATAAG
Angiotensin - Reverse	AATTCTTATTACAGATGAAACGGATGAATATACACCCGGTCC
SubstanceP - Forward	GATCGCGGCCGAAACCGCAACAGTTCTTTGGCCTGATGTAATAAG
SubstanceP - Reverse	AATTCTTATTACATCAGGCCAAAGAACTGTTGCGGTTTCGGCCGC
Gastrin - Forward	GATCGCAGGGTCCGTGGCTGGAAGAAGAAGAAGAAGCGTATGGCTGGA TGGACTTCTAATAAG
Gastrin - Reverse	AATTCTTATTAGAAGTCCATCCAGCCATACGCTTCTTCTTCTTCTTCCAGC CACGGACCCTGC
Katacalcin - Forward	GATCGGACATGAGCAGCGATCTGGAACGCGATCATCGCCCGCATGTGAG CATGCCGCAGAACGCGAACTAATAAG
Katacalcin - Reverse	AATTCTTATTAGTTCGCGTTCTGCGGCATGCTCACATGCGGGCGATGATC GCGTTCCAGATCGCTGCTCATGTCC
**PCR primers for hGH isoform 1 to be fused at the C-terminal of IgG_1 Fc**	
Forward	TTTTTTTTGGATCCTTCCCAACCATTCCCTTATC
Reverse	TTTTTTTTGAATTCTTATTAGAAGCCACAGCTGCCCTCC
**PCR primers for IgG_1 C_H3 (C250A, C308A) domain**	
Forward	TTTTTTTTCATATGGGTCAGCCGCGGG
Reverse	TTTTTTTTGAATTCTTATTAGGATCCTTATTAGTGATGGTGATGGTGATG
**PCR primers for IgG_1 C_H2 domain with the hinge region (T106-A222)**	
Forward	TTTTTTTTCATATGACCCATACCTGCCCGC
Reverse	TTTTTTTTGGATCCTTATTACGCTTTGCTAATGGTTTTTTCAATCGGCG
**PCR primers for IgG_1 C_H2 domain without the hinge region (G119-A222)**	
Forward	TTTTTTTTCATATGGGCGGCCCGAGC
Reverse	TTTTTTTTGGATCCTTATTACGCTTTGCTAATGGTTTTTTCAATCGGCG

2. Accession numbers of the protein sequences used in this study:

Human IgG1 Fc region: P01857

Trebananib: 894356-79-7

Leptin: P41159

Human growth hormone Isoform 1: B1A4G6

Angiotensin: P01019

Substance P: P20366

Gastrin: P01350

Katacalcin: P01258
